# Draft Genome Sequences of Two Heat-Resistant Mutant Strains (A52 and B41) of the Photosynthetic Hydrogen-Producing Bacterium *Rhodobacter capsulatus*

**DOI:** 10.1128/genomeA.00531-16

**Published:** 2016-06-09

**Authors:** Abdulmecit Gokce, Zeynep Petek Cakar, Meral Yucel, Orhan Ozcan, Sevde Sencan, Ibrahim Sertdemir, Bekir Erguner, Betul Yuceturk, Aydan Sarac, Bayram Yuksel, Yavuz Ozturk

**Affiliations:** a Department of Molecular Biology and Genetics, Faculty of Science & Letters, Istanbul Technical University, Istanbul, Turkey; b Department of Molecular Biology and Genetics, Faculty of Engineering and Natural Sciences, Bahcesehir University, Istanbul, Turkey; c Istanbul Technical University, Dr. Orhan Ocalgiray Molecular Biology, Biotechnology & Genetics Research Center, Istanbul, Turkey; d Department of Biology, Middle East Technical University, Ankara, Turkey; e TUBITAK Marmara Research Center, Genetic Engineering and Biotechnology Institute, Kocaeli, Turkey; f TUBITAK, National Research Institute of Electronics and Cryptology, Advanced Genome & Bioinformatics Research Laboratory, Kocaeli, Turkey

## Abstract

The draft genome sequences of two heat-resistant mutant strains, A52 and B41, derived from *Rhodobacter capsulatus* DSM 1710, and with different hydrogen production levels, are reported here. These sequences may help understand the molecular basis of heat resistance and hydrogen production in *R. capsulatus*.

## GENOME ANNOUNCEMENT

*Rhodobacter capsulatus* is a purple alphaproteobacterium known for its metabolic plasticity, enabling it to thrive in disparate ecological niches (1, 2). Furthermore, this feature of *R. capsulatus* makes it an ideal model to study respiratory, photosynthetic, and chemolithotrophic growth modes (1–3). In the last two decades, studies on *R. capsulatus* particularly focused on the ability of this organism to produce hydrogen (3–5). In order to achieve sustainable hydrogen production in outdoor photobioreactors, two heat-resistant mutant strains (A52 and B41) of *R. capsulatus* DSM 1710 have been developed by our group through a directed evolution approach, using chemical mutagenesis (5).

In this study, the genomes of two heat-resistant mutant strains of *R. capsulatus* (A52 and B41), with different hydrogen production levels, were sequenced using the Illumina HiSeq 2000 platform. Paired-end sequencing was conducted with two different insert sizes of 500 and 2,000 bp. Filtering of the raw data for low-quality bases, adapter contamination, and duplicates resulted in a clean sequence, providing 180-fold coverage for both of the draft genomes. The reads were assembled with A5-miseq pipeline version 20141120 (6). The A52 and B41 genome assemblies produced 70 and 64 scaffolds, with *N*_50_ values of 164,800 and 182,811 bp, respectively. The maximum read length of 430 kb was obtained for both genomes, with a total length of 3.67 Mb of data.

The NCBI Prokaryotic Genome Annotation Pipeline was used for prediction and annotation of the draft genome sequences (7). The resultant draft genome sequences had a mean G+C content of 66.5% for 3,483 genes, 3,350 coding sequences (CDSs), 79 pseudogenes, 50 tRNAs, and 1 of each 5S, 16S, and 23S rRNAs, for both A52 and B41. The SEED viewer version 2.0 was used to categorize the predicted genes into functional subsystems (8). Of the coding sequences, 50% were assigned to subsystems. Putative functions were assigned to 2,592 protein-coding genes, whereas 907 hypothetical proteins had no match to any known proteins. Whole-genome comparison of the draft genomes revealed a close relationship to *R. capsulatus* SB1003, with a 500 similarity score on RAST (genome ID 272942.6) (8).

These genome sequences of *R. capsulatus* A52 and B41 will serve as important references for further studies focusing on their heat resistance and the genes that enable hydrogen production, and for further comparative studies with other alphaproteobacteria.

### Nucleotide sequence accession numbers.

These whole-genome shotgun projects have been deposited at DDBJ/EMBL/GenBank under the accession numbers LLVU00000000 and LLVV00000000 for B41 and A52, respectively. The versions described in this paper are versions LLVU00000000.1 and LLVV00000000.1.
